# Safety-constrained evaluation of antibacterial multicomponent borate glasses

**DOI:** 10.1016/j.bbiosy.2026.100140

**Published:** 2026-05-14

**Authors:** Christine Andrea, Daniel Boyd

**Affiliations:** a School of Biomedical Engineering, Dalhousie University, Halifax B3H 4R2, NS, Canada; b Department of Applied Oral Sciences, Faculty of Dentistry, Dalhousie University, 5981 University Avenue, Halifax B3H 4R2, NS, Canada

**Keywords:** Bioactive glass, Ion release kinetics, Antibacterial materials, Toxicological risk assessment, Design of mixtures, Toothpaste materials, Dental caries prevention

## Abstract

Soluble ion-releasing materials intended for transient, dilution-limited use must achieve functional performance while maintaining safety under repeated exposure. This study presents a clinically parameterized framework for the design of multi-ion, soluble glasses for toothpaste applications by integrating a Design-of-Mixtures composition space with brushing-relevant extraction conditions. Glasses within a constrained Ag_2_O–NaF–CaO design domain were synthesized, characterized, and evaluated using 2 min aqueous extracts to quantify dissolution behavior, multi-element ion release, and antibacterial activity against *Streptococcus mutans* at clinically plausible dilutions. By maintaining a constant extraction ratio across the mixture space, compositional effects on early-time dissolution and ion speciation were isolated under exposure conditions relevant to transient plaque pH challenges. Composition–response modeling demonstrated that calcium release establishes a thermodynamically constrained, remineralization-relevant baseline, whereas silver-mediated antibacterial activity is non-monotonic and highly composition-dependent, indicating that antibacterial potency can be engineered through controlled dissolution rather than increased silver loading. Ag_2_O–NaF interaction effects consistent with speciation-sensitive co-release were identified, supporting strategies that preserve fluoride availability while optimizing antimicrobial performance. An ISO 10993-17-aligned toxicological risk assessment was applied as a conservative, exposure-based screening tool to map efficacy–safety relationships across the design space and prioritize dose-efficient compositions. Together, this workflow provides a safety-constrained framework for evaluating antibacterial ion-releasing glasses and for assessing transient, multi-ion delivery materials under realistic exposure conditions.

## Highlights

• Exposure-anchored framework evaluates ion-releasing biomaterials under realistic use.
• Design-of-Mixtures resolves composition–interaction effects on functional properties.
• Antibacterial efficacy is non-monotonic and tunable without dose escalation.
• Safety positioned as a primary design variable via ISO 10993-17 risk mapping.
• Defines a transferable safety–efficacy window for transient ion delivery systems.

## 1 Introduction

Dental caries remains the most prevalent non-communicable disease worldwide, affecting billions across the lifespan and imposing a substantial clinical and economic burden on global health systems [1,2]. Contemporary understanding increasingly frames dental caries as a biofilm-mediated, diet-driven dysbiosis rather than an acute infectious process [[3], [4], [5]]. Disease progression is governed by repeated, short duration acidogenic challenges following carbohydrate intake, which shift the balance between demineralization and remineralization within dental biofilms [6,7]. This paradigm shift has important implications for preventive strategies: effective interventions must operate within brief, repeatedly applied exposure windows rather than relying on prolonged antimicrobial contact [8,9].

Toothpaste occupies a unique position within this preventive framework. It is applied daily and at population scale, yet its therapeutic window is tightly constrained by the mechanics of brushing [10]. Typical brushing events last approximately two minutes and are accompanied by substantial dilution due to stimulated salivary flow, resulting in a transient, high-dilution environment in which active agents must act rapidly to exert meaningful biological effects [[11], [12], [13], [14]]. This narrow therapeutic window exposes a fundamental limitation of many conventional material designs, which rely on bulk properties, sustained release, or prolonged surface contact poorly suited to the rapid, transient oral environment. Under these conditions, efficacy is governed by early-time interactions between materials and the oral environment, favoring approaches capable of delivering immediate, localized effects upon contact within this narrow exposure window [15,16].

These constraints have sharpened interest in antibacterial strategies aligned with antimicrobial stewardship principles. While dental caries is not a systemic infection, dysbiotic oral biofilms contribute meaningfully to outpatient antibiotic prescribing, positioning preventive oral healthcare as an upstream lever to reduce unnecessary antimicrobial exposure and resistance burden [[17], [18], [19]]. In this context, dose-efficient, non-antibiotic approaches that suppress early bacterial outgrowth without imposing excessive selective pressure are increasingly emphasized by global health agencies [20]. Materials capable of delivering rapid, localized, multi-mechanistic antibacterial action under short-contact conditions thus represent a compelling alternative to conventional antimicrobials designed for prolonged or high-dose exposure, provided therapeutic function is intrinsically embedded within the material to maximize efficacy while minimizing systemic exposure and resistance development [[21], [22], [23]].

A wide range of material-based strategies has been explored for dental caries prevention, including conventional antimicrobial agents, remineralizing additives, and ion-releasing biomaterials. Nevertheless, many of these approaches are optimized for prolonged contact or sustained local concentration, limiting their effectiveness under the transient conditions of toothbrushing [5,8]. Within this landscape, bioactive glasses have attracted increasing attention for their ability to deliver therapeutic inorganic ions through controlled dissolution, enabling simultaneous antibacterial and remineralization-relevant effects without reliance on conventional antibiotics [[24], [25], [26], [27]]. While silicate-based bioactive glasses have been widely explored for hard-tissue repair, borate-based systems are distinguished by their intrinsically higher dissolution rates and composition-dependent early ion release behavior [28,29]. Consistent with this higher reactivity, borate-based bioactive glasses have often shown a more pronounced early in vitro cytotoxic response than silicate-based systems, reflecting faster degradation and higher initial ion flux under static assay conditions [28]. In borate networks, the absence of a passivating silica-rich layer permits rapid water penetration and bulk hydrolysis, enabling near-congruent ion release on brushing-relevant timescales measured in minutes rather than hours [[30], [31], [32]]. Consistent with this rapid-release profile, borate-containing bioactive glasses have been incorporated into experimental oral care formulations, including dentifrice systems evaluated in preclinical and early clinical contexts, underscoring their translational feasibility [33,34].

Despite these advantages, multicomponent borate bioactive glasses have not been systematically designed or evaluated under clinically realistic, short-duration exposure conditions relevant to daily oral hygiene. Prior studies, by predominantly examining materials under prolonged immersion conditions or through single-ion and isolated biological endpoints, have left unresolved the integrated challenge of achieving rapid antibacterial efficacy, remineralization, and acceptable systemic safety within the narrow exposure window imposed by toothbrushing [27,[35], [36], [37]]. Within this exposure framework, toxicological risk assessment (TRA) provides a quantitative means to treat safety as a composition-dependent design constraint rather than a post hoc outcome, enabling measured ion release to be directly compared against established tolerable intake thresholds through exposure-based safety frameworks [38]. In parallel with safety considerations, the biological functionality of borate bioactive glasses derives from the coordinated release of calcium, silver, and fluoride, whose distinct roles in mineral thermodynamics, antibacterial activity, and mineral stabilization are intrinsically coupled through network structure and dissolution behavior in multicomponent systems [9,33,[39], [40], [41], [42]].

Accordingly, this study aims to systematically investigate multicomponent borate bioactive glass compositions. Using a Design-of-Mixtures (DoM) framework, we evaluate performance under a clinically informed two-minute extraction regime, representative of toothbrushing conditions. Safety is assessed via an ISO 10993–aligned toxicological risk assessment, while efficacy is determined through early dissolution, multi-ion release, and antibacterial response against *S. mutans*. The objective of this work is to delineate a therapeutic window where optimal antibacterial efficacy and remineralization-relevant calcium availability coexist with acceptable margins of systemic safety, thereby establishing a rational basis for the design of dose-efficient bioactive materials for preventive oral healthcare.

## 2 Methods

Sixteen borate glass compositions (BAGF) were designed using a Design-of-Mixtures (DoM) statistical framework (Design-Expert v13.0.5, Stat‑Ease Inc., USA) [43]. The mixture design constraints defining the compositional space explored for BAGF glass formulations included the following components: B_2_O_3_ ranged from 73 to 84 mol%, CaO ranged from 5 to 10 mol%, Ag_2_O ranged from 0 to 1.5 mol%, NaF ranged from 5 to 10 mol%, and Na_2_SO_4_ was fixed at 5 mol%. Elemental ranges of these compositions can be seen in Table 1. Glass synthesis, processing, and physicochemical characterization were performed as previously described [44,45].

**Table 1:** Elemental ranges of BAGF compositions, calculated daily ion masses used for toxicological risk assessment (Expected Exposure), and Margin of Safety (MOS) values calculated for ranges of boron (20 mg/day), fluoride (3.5 mg/day), and silver, where C indicates conservative loading of silver (0.35 mg/day) and P indicated permissive loading of silver (1.0 mg/day), assuming full-dissolution daily exposure conditions.

Ingredient	Composition by Weight (%)		Daily Expected Exposure (EE) (mg/day)		Margin of Safety (MOS)	
	Min	Max	Min	Max	Min	Max
Boron	22.22	25.15	7.44	8.42	2.4	2.7
Calcium	2.77	5.76	0.93	1.93		
Fluorine	1.30	2.73	0.43	0.91	3.8	8.1
Oxygen	56.37	62.05	18.88	20.79		
Silver	0	4.52	0	1.51	0.2^C^	1.5^C^
					2.2^P^	14.3^P^
Sodium	4.70	6.61	1.58	2.21		
Sulfur	2.19	2.30	0.73	0.77		

### 2.1 Extract ratio

A clinically relevant extraction ratio corresponding to a 2 min brushing duration was defined using a toothpaste mass of 0.67 g per use, derived from American Dental Association guidance and published measurements of toothpaste mass per brushing [11,46,47]. At a formulation target of 5 wt% BAGF, this corresponds to 33.5 mg of BAGF introduced intra-orally per use, consistent with typical toothpaste additive loadings and sufficient for quantifiable ion release under 2 min extraction. To estimate the available dilution volume, stimulated salivary flow during brushing was quantified using clinical and literature inputs. Independent studies reported flow rates of 3.23 mL/min [48] and 3.5 mL/min [49], while clinician consultation indicated that toothpaste typically elicits flow toward the upper end of the 1–3 mL/min physiological range. A value of 3.25 mL/min was therefore selected as representative of brushing-induced salivation. Over a 2 min brushing duration, this corresponds to:

TotalSalivaVolume=3.25mL/min×2min=6.5mL

The clinically relevant extract ratio was defined as:

$$\text{Extract}\;\text{Ratio}=\frac{33.5\;\text{mg}\;\text{BAGF}}{6.5\;\text{mL}\;\text{saliva}}\approx 5.15\;\text{mg}/\text{mL}$$

Accordingly, all 2 min dissolution, ion-release, and antibacterial assays were performed at an extraction ratio of 5.15 mg/mL.

### 2.2 Initial toxicological risk assessment (TRA)

A theoretical toxicological risk assessment (TRA) was performed following ISO 10993-17 (Toxicological Risk Assessment of Medical Device Constituents) to evaluate potential systemic exposure arising from short-duration, ingestion dominated contact associated with toothpaste use [38]. Adult daily use was modelled as 0.67 g toothpaste, containing 5 wt% bioactive glass (BAGF), corresponding to 33.5 mg glass/day. Exposure was assumed to occur via episodic, transient oral contact (∼2 min per use) with subsequent swallowing of dissolved ions, rather than prolonged mucosal contact. Elemental masses for boron, calcium, fluoride, oxygen, silver, sodium, and sulfur were calculated from the measured compositional ranges (Table 1). Essential elements present at levels below their established dietary recommended intakes (DRIs), including calcium (1000 mg/day), sodium (1500 mg/day), as well as sulfur, which is abundant in the human diet and exhibits low toxicity at anticipated exposure levels, were not further assessed [[50], [51], [52]]. Oxygen is present as oxide in the glass network and does not constitute an exposure-relevant constituent for systemic toxicity in this context. Accordingly, the TRA focused on boron, fluoride, and silver as potential risk-driving ions.

For boron, the Tolerable Upper Intake Level (UL) established by the Institute of Medicine, 20 mg/day, was used as the Tolerable Intake (TI) [53,54]. For fluoride, the Adequate Intake (AI) values for adult males (4 mg/day) and females (3 mg/day) were used directly as Tolerable Exposure (TE) values, consistent with ISO guidance. Because AIs already incorporate uncertainty factors and concomitant exposure considerations, an average adult TE of 3.5 mg/day was applied in this assessment [51,55]. For silver, the U.S. Environmental Protection Agency (EPA) Reference Dose (RfD) of 0.005 mg/kg•day was used as the conservative TI, with a 70-kg adult body weight applied to derive TE; a secondary, permissive TE of 1 mg/day, derived from human observational data, was included for sensitivity analysis [[56], [57], [58], [59], [60]]. TE values (mg/day) were calculated using the ISO 10993-17 utilization factor (UTF) framework [38]:

$$TE=TI\times m_{B}\;x\;CEF\;x\;PEF$$

where m_B_​ is adult body weight (70 kg), the Concomitant Exposure Factor (CEF) was set to 1.0 for the general population, and the Patient Exposure Factor (PEF) was set to 1.0 for chronic daily toothpaste use. Because fluoride TE is defined by AI values, the UTF was not applied. For each ion and each BAGF composition, a Margin of Safety (MOS) was calculated as:

$$MOS=\frac{TE}{EE}$$

where EE is the expected daily exposure assuming worst-case 100% dissolution and 100% ingestion of the ion mass released from 33.5 mg BAGF/day. A MOS ≥ 1 was considered acceptable and indicative of low toxicological concern, consistent with ISO 10993-17.

### 2.3 Dissolution and mass loss assessment

All dissolution experiments were conducted in triplicate under sterile conditions within a Class II biosafety cabinet (BSC) to ensure that the resulting extracts were suitable for downstream microbiological analysis. HEPES buffer (0.05 M, pH 7.6) was prepared using Type I water, and the pH meter was calibrated immediately prior to preparation. The solution was subsequently vacuum-filtered through a 0.2 µm P membrane into sterile, foil-wrapped containers to minimize photochemical degradation, as HEPES is known to exhibit light sensitivity, particularly in the presence of metal ions. Filtered buffer was stored at 4 °C when not in use. An aliquot of sterile HEPES was reserved as a negative control for both ion-release quantification and antibacterial assays.

Three sterile tube types were used and organized according to their downstream analytical purposes: Tube A for dissolution and mass-loss measurements, Tube B for sterile-filtered extracts used in ion-release analysis, and Tube C for sterile-filtered extracts used in MIC/MBC testing. For each replicate, 0.0515 g of BAGF powder (<45 µm) was weighed into sterile scintillation vials. Corresponding Tube A containers were pre-weighed (tube + cap), labeled, and transferred into the BSC. Within the BSC, the weighed glass was transferred into Tube A and combined with 10 mL of sterile, filtered HEPES. Tubes were then manually inverted for 2 min at 50 inversions per 30 s (∼1.67 Hz), a frequency selected to approximate the hydrodynamic shear imparted by orbital shaking at 100–120 rpm. Throughout dissolution, tubes were protected from direct light to mitigate photochemical artifacts associated with HEPES, particularly in the presence of silver-containing glass compositions.

Immediately after the 2 min inversion, tubes were sealed and centrifuged (4400 x g, RCF) for 1 min to sediment residual particulates. The clarified supernatant was then recovered within the BSC using sterile serological pipettes. A total of 10 mL of supernatant was passed through a 0.2 µm syringe filter into Tube B, yielding sterile extracts appropriate for subsequent analyses. From Tube B, 5 mL aliquots were transferred into Tube C, and triplicate aliquots were combined to obtain 15 mL of sterile extract for MIC/MBC testing. All tubes were sealed with sterile Parafilm®, stored at 4 °C, and protected from light until further analysis. After supernatant removal, Tube A containing the residual pellet was transferred to a drying oven and held at 50 °C for > 48 h. Tubes were cooled and re-weighed to determine the final mass. The percentage mass loss was calculated as follows:

$$\%\;Mass\;Loss=\;\frac{m_{initial}-\;m_{final}}{m_{initial}}\;x\;100$$

where *m*_initial_ is the pre-incubation mass (Falcon tube and cap, glass sample) and *m*_final_ is the final post-drying mass (Falcon tube and cap, remaining glass sample). Strict adherence to timing between inversion, centrifugation, and supernatant removal was critical to avoid over-estimating dissolution during sample handling.

### 2.4 Ion release analysis

To quantify the concentration of therapeutic ions in the extracts (Ag and Ca), inductively coupled plasma–optical emission spectroscopy (ICP-OES; Optima DV8000, PerkinElmer, USA) was conducted. Following dissolution, 5 mL aliquots of each sterile extract were stored at 4 °C without acidification to preserve ionic equilibria. Prior to analysis, samples were diluted 10X or 100X, depending on expected concentration ranges determined during pilot testing. ICP-OES measurements were performed using a 2% HNO₃ carrier matrix, N₂ carrier gas, and an argon plasma. Calibration curves (0–5 ppm) were prepared using certified multi-element standards (PerkinElmer, Canada), and samples were further diluted as necessary to remain within the calibrated detection range. All measurements were conducted in triplicate, and final ion concentrations were corrected to reflect the 10 mL extraction volume used in the dissolution step.

### 2.5 Antibacterial testing

Antibacterial activity of BAGF extracts was assessed against *Streptococcus mutans* (ATCC® 25175™) using a broth microdilution method adapted from the procedures performed by Perfectus Biomed (NAMSA). Forty-eight–hour cultures of *S. mutans* grown on Brain Heart Infusion agar supplemented with 5% horse blood (BHIHBA) were harvested and suspended in double-strength Brain Heart Infusion broth (BHIB) to obtain an inoculum of 1 × 10^8^ ± 5 × 10^7^ CFU/mL. This suspension was diluted in double-strength BHIB to yield a working inoculum of 1 × 10^5^ ± 5 × 10^5^ CFU/mL, generating a final target concentration of 5 × 10^5^ ± 3 × 10⁵ CFU/mL upon 1:1 mixing with BAGF extracts in the minimum inhibitory concentration (MIC) assay. The inoculum was verified by performing serial 10-fold dilutions in phosphate-buffered saline (PBS) and plating onto BHIHBA, followed by incubation at 37 ± 2 °C with 5% CO₂ for 48 h.

Extracts were dispensed into sterile, tissue-culture–treated 96-well flat-bottom microplates and serially 2-fold diluted in sterile HEPES buffer (0.05 M, pH 7.6). MIC plates were prepared by adding 100 μL of the standardized inoculum to 100 μL of each extract dilution. Plates were incubated for 24 h at 37 ± 2 °C under ambient atmospheric conditions. Sterility controls (broth only), growth controls (broth + inoculum), and buffer controls (HEPES only) were included in each run.

Bacterial growth was monitored by measuring optical density at 600 nm (OD₆₀₀) using a Varioskan™ Lux microplate reader (Thermo Fisher Scientific, USA). Absorbance readings were recorded at 0, 4, 8, 12, 16, 20, and 24 h to characterize inhibition kinetics and identify concentrations capable of suppressing visible growth (MIC). At the 24-hour endpoint, the contents of wells from each extract dilution were plated onto BHIHBA to distinguish true growth inhibition from optical suppression and to evaluate bactericidal activity. Plates were incubated at 37 ± 2 °C with 5% CO₂ for 48 h, after which the presence or absence of colony formation was recorded. Each extract concentration was tested in triplicate. MIC was defined as the lowest extract dilution showing no visible growth, as evidenced by absence of turbidity and no increase in OD₆₀₀ relative to the broth + inoculum control after 24 h. MBC was defined as the lowest extract dilution yielding no recoverable colonies upon subculture onto BHIHBA, corresponding to a ≥ 99.9% reduction in viable colony forming units (CFU).

### 2.6 Statistical analysis

Experimental responses across the compositional design space were analyzed using the DoM framework in Design-Expert [43]. Reduced quadratic mixture regression models were fit to dissolution, ion release, and antibacterial response data using mean values from replicate measurements, where applicable. Actual equations were used for response prediction, while coded equations were used to interpret the relative effects of components and their interactions within the design space. Model significance and term effects were evaluated by analysis of variance (ANOVA), and model adequacy was assessed using R^2^, adjusted R^2^, predicted R^2^, and adequate precision. Models with limited predictive strength were retained only for qualitative trend interpretation where signal-to-noise was sufficient, rather than for quantitative optimization.

## 3 Results

Detailed analyses of the BAGF compositions evaluated in this study, including structural, physical, and chemical properties, as well as dissolution and multi-ion release behavior under physiological conditions, are documented elsewhere [44,61]. Notably, all glasses were processed to achieve a uniform particle size distribution (<45 µm), ensuring consistent surface area–to–volume ratios across compositions. This uniformity facilitates direct comparison of early dissolution, antibacterial performance, and toxicological outcomes evaluated in this study.

### 3.1 Theoretical toxicological risk assessment (TRA)

Daily ion masses derived from full dissolution of 33.5 mg BAGF/day were used to calculate EE values for boron, fluoride, and silver (Table 1). Calcium, sodium, sulfur, and oxygen exposures were below their corresponding dietary or toxicological relevance thresholds and were therefore not assessed further. MOS values were computed for each ion based on their respective TEs. Across the full boron compositional range (22.22–25.15 wt%), EE values yielded MOS values > 1 for all BAGF formulations (Table 1). The minimum and maximum MOS values indicate that daily boron exposure remains well below the adult UL of 20 mg/day, even under the conservative assumption of complete dissolution. Boron therefore does not limit the systemic safety profile of any formulation in the design space. Fluoride EE values, reflecting 1.30–2.73 wt% F across compositions, were compared to the averaged adult TE of 3.5 mg/day. All formulations demonstrated MOS > 1, with MOS values ranging from 3.8 to 8.1 (Table 1). These data confirm that fluoride release from BAGF, at the modelled loading and use pattern, does not approach systemic toxicity thresholds and is not a constraining factor for formulation safety.

Silver content varied across four composition groups (0 mol%, 0–0.75 mol%, 0.75–1.5 mol%, and ≥1.5 mol%). As expected from the relatively low toxicity thresholds for silver intake, silver exhibited the lowest MOS values and thus served as the primary risk-driving ion. Under the conservative TE, formulations within 0–0.75 mol% Ag₂O displayed MOS ≥ 1, indicating acceptable systemic risk (Table 1). However, MOS values for formulations above approximately 1.0 mol% Ag₂O fell below 1, suggesting that high-silver compositions exceed conservative daily intake limits under the full-dissolution scenario. Under the permissive TE, all formulations except those containing ≥1.5 mol% Ag₂O exhibited MOS ≥ 1, with MOS values ranging from 2.2 to 14.3. This represents a substantially expanded safety envelope compared to the RfD-based TE. Overall, silver defines the upper boundary of the systemic safety window, with acceptable exposure depending on both the Ag₂O content and the TE framework applied.

### 3.2 Dissolution

**Fig. 1:**
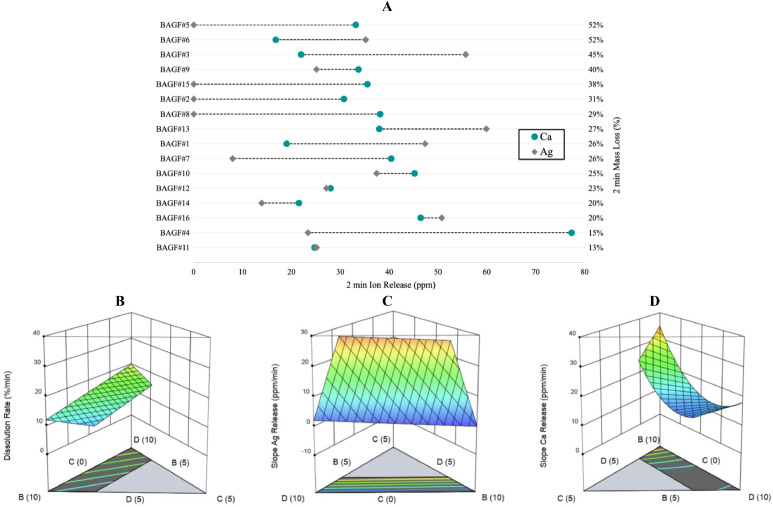
(A) Distribution of 2 min dissolution (%) and ion release (ppm) values for BAGF compositions, illustrating the overall range and variability across the design space. DoM-derived 3D response surface showing the influence of CaO (“B”), Ag2O (“C”), and NaF (“D”) (mol%) on (B) dissolution rate, (C) release rate of silver, and (D) release rate of calcium, calculated from the slope at the 2 min time point (%/min and ppm/min.

All glasses exhibited measurable dissolution in HEPES buffer at the selected extract ratio (5.15 mg/mL), with 2 min mass loss values ranging from 12.9 to 52.3%. The resulting extract concentrations calculated from the measured mass loss therefore ranged from 0.67 to 2.69 mg/mL. Corresponding dissolution rates, derived from the slope of mass loss (%) regressed against time, ranged from 6.5 to 26.1%/min. The distribution of early dissolution behavior is shown in Fig. 1A, illustrating the variability in 2 min mass loss across compositions. A reduced quadratic regression yielded statistically robust predictors for the dissolution slope (Table 2). Among the compositional terms, NaF exhibited the largest positive effect, whereas Ag₂O showed the strongest negative effect. In coded form, positive contributors to 2 min mass loss were NaF > B₂O₃, while B₂O₃*NaF > Ag₂O > CaO were negative contributors, with the B₂O₃*NaF interaction exhibiting the greatest overall influence (Fig. 1B).

**Table 2:** Reduced quadratic regression models (actual equations) generated using the DoM framework for dissolution rate (%/min) and ion release rate responses (ppm/min), and antibacterial inhibition based on 0–2 min data, with associated model fit statistics. Models with R^2^ < 0.5 are retained as trend-level fits (see footnote†).

Response	Regression Model	R^2^	R^2^	R^2^ Predicted	Adequate Precision
			Adjusted		
Dissolution Rate	1.21 * B_2_O_3_ - 6.21 * CaO - 7.54 * Ag_2_O + 47.05 * NaF - 0.66 * B_2_O_3_*NaF	0.67	0.55	0.30	8.55
Ag Release Rate	0.11 * B_2_O_3_ - 0.74 * CaO + 16.44 * Ag_2_O - 0.29 * NaF	0.89	0.86	0.83	14.81
Ca Release Rate	–0.07 * B_2_O_3_ + 76.07 * CaO - 5.99 * Ag_2_O + 6.47 * NaF - 0.83 * B_2_O_3_*CaO - 1.82 * CaO*NaF	0.74	0.62	0.33	9.97
% *S. Mutans* Inhibition	–0.90 * B_2_O_3_ - 212.46 * CaO + 30.95 * Ag_2_O + 152.23 * NaF + 2.69 * B_2_O_3_*CaO - 1.74 * B_2_O_3_*NaF + 4.28 * CaO*Ag_2_O - 5.03 * Ag_2_O*NaF	0.91	0.84	0.51	11.19

† Models with R^2^ < 0.5 were retained as trend-level fits due to adequate precision ratios >4, indicating sufficient signal-to-noise to support qualitative navigation of the composition space. Low adjusted and predicted R^2^ values indicate limited predictability utility; fitted surfaces are therefore presented for qualitative trend visualization rather than quantitative optimization.

### 3.3 Ion release analysis

**Fig. 2:**
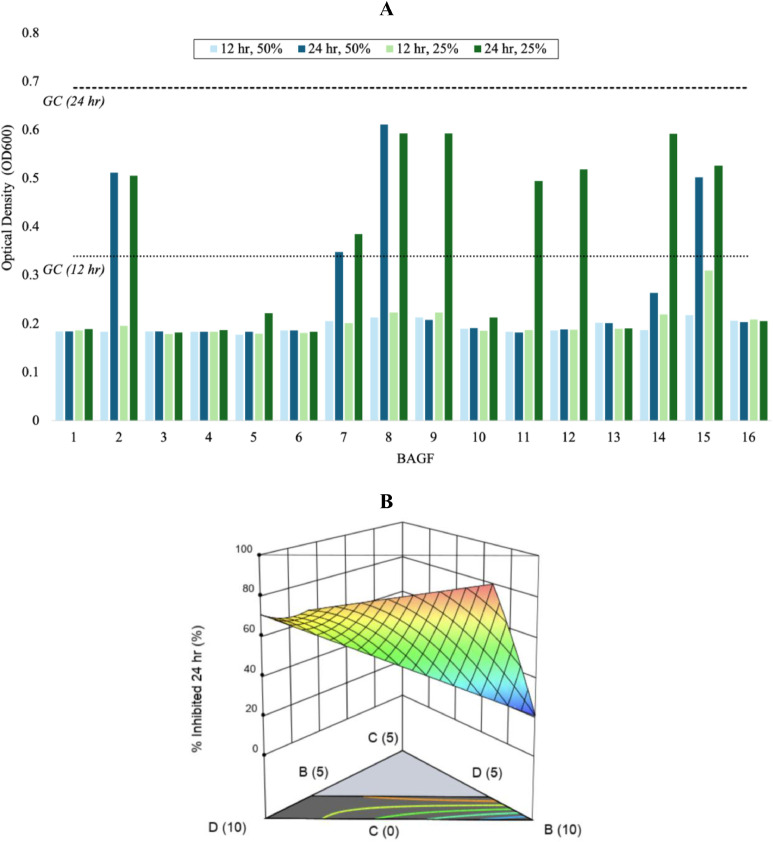
(A) Optical density profiles of S. mutans following exposure to BAGF extracts at 50% and 25% after 12 h and 24 h incubation. Bars represent mean OD_600_ values from n = 3 replicates and illustrate qualitative trends across compositions and extract conditions. Dashed horizontal lines denote the mean growth control (GC) OD values at 12 and 24 h, with plate-specific control conditions averaged to allow comparison across assay plates. Antibacterial classification based on OD response and agar recovery identified BAGF 1, 3, 6, and 13 as bactericidal (MBC ≤ 50%); BAGF 4, 5, 9, 10, 11, and 16 as bacteriostatic (MIC observed, MBC > 50%); and BAGF 2, 7, 8, 12, 14, and 15 as no effect under the conditions tested. MIC was defined as the lowest concentration with no increase in optical density, and MBC as the lowest concentration with no recoverable colonies after 24 h. (B) DoM-derived 3D response surface showing the influence of CaO (“B”), Ag_2_O (“C”), and NaF (“D”) (mol%) on the percent inhibition of S. mutans, calculated from OD-based growth suppression.

Across the glass series, the two-minute release values for silver and calcium varied, with calcium release showing a narrower range and remaining detectable for all compositions. In contrast, silver release displayed a broader distribution and greater variability between compositions (Fig. 1A). At 2 min, silver concentrations ranged from 0 to 60 ppm, with release rates between 0 and 30 ppm/min. Reduced quadratic mixture regression models showed an excellent fit for silver release rates (R^2^=0.89, Table 2). In the actual-coefficient model, Ag₂O emerged as the dominant compositional term affecting silver release, while the coded equation analysis ranked positive effects as Ag₂O > B₂O₃, with CaO > NaF having negative contributions. Calcium concentrations at 2 min ranged from 16.8 to 77.3 ppm, with release rates between 8.4 and 38.7 ppm/min. In the actual-coefficient model, CaO was identified as the dominant compositional term influencing calcium release (Table 2), while the coded equation analysis ranked positive effects as CaO > NaF > B_2_O_3_, with CaO*NaF > B_2_O_3_*CaO > Ag_2_O contributing negatively. The interaction of CaO*NaF had the most significant influence in the coded equations. Representative three-dimensional response surfaces illustrating these compositional effects for both silver and calcium release rates are shown in Figs. 1C and 2D.

### 3.4 Antibacterial activity

Based on combined MIC and MBC outcomes, four compositions (BAGF 1, 3, 6, and 13) exhibited bactericidal activity, evidenced by the absence of recoverable colonies at the highest tested extract concentration (MBC ≤ 50%). Six compositions (BAGF 4, 5, 9, 10, 11, and 16) exhibited bacteriostatic behavior, indicated by reduced optical density with continued viable growth on agar. The remaining six formulations (BAGF 2, 7, 8, 12, 14, and 15) showed no discernible antibacterial effect, with growth observed at all tested concentrations.

Optical density measurements further elucidated time- and concentration-dependent growth trends (Fig. 2A). At 12 h, OD_600_ values for most BAGF extracts were lower than those of the corresponding growth controls at both 50% and 25% extract concentrations. By 24 h, a clear divergence emerged among the activity groups. Bactericidal and bacteriostatic compositions maintained low OD values at 50% extract, whereas compositions with no effect exhibited notable growth recovery, particularly at 25% extract, where OD values approached those of the growth controls. These data suggest that growth suppression at 12 h did not consistently persist to 24 h across all compositions.

To facilitate quantitative comparison across the compositional design space, percent inhibition at 24 h was calculated from OD data normalized to plate-specific growth controls and modeled using reduced quadratic mixture regressions (Table 2). The resulting models provided good agreement with experimental inhibition values and were used to generate both actual equations for prediction and coded equations for assessing relative factor contributions. Coded-equation analysis indicated that CaO*Ag_2_O > B_2_O_3_*CaO > Ag_2_O > NaF > B_2_O_3_ contributed positively to the inhibition response, whereas Ag_2_O*NaF > B_2_O_3_*NaF > CaO had negative effects, with the interaction of Ag_2_O*NaF exerting the highest magnitude of influence. The corresponding 3D response surface (Fig. 2B) visualizes the non-linear variation in antibacterial performance across the mixture space, highlighting distinct regions of elevated and reduced inhibition.

### 3.5 Correlations and integration of safety and efficacy

**Fig. 3:**
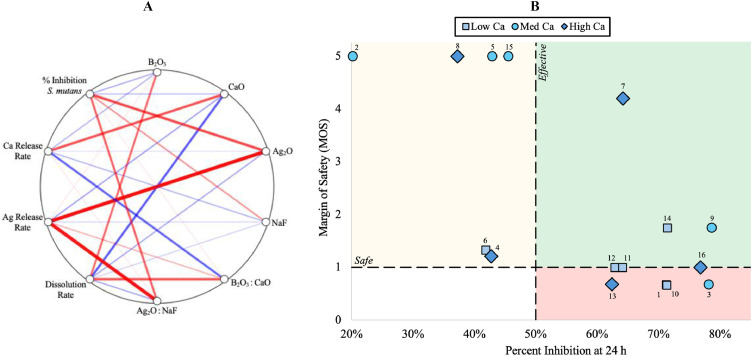
(A) Circular correlation plot illustrating relationships between early dissolution kinetics, ion release, and antibacterial efficacy as a function of composition and key component ratios. Lines denote Pearson correlation coefficients (r) linking response variables to compositional predictors, with line thickness and intensity scaling with ∣r∣. Red and blue lines indicate positive and negative correlations, respectively. (B) Therapeutic window mapping of antibacterial efficacy and margin of safety across BAGF compositions. Percent inhibition of S. mutans at 24 h is plotted against margin of safety (MOS), derived from silver TRA data. Dashed lines indicate the efficacy threshold (> 50% inhibition) and the safety threshold (MOS > 1). Shaded regions denote unsafe (red), safe but weak (yellow), and safe and effective (green) compositions. Marker shape reflects relative calcium release rates: low (8–15 ppm/min), medium (15–20 ppm/min), and high (20–40 ppm/min). Silver-free compositions (MOS → ∞) are plotted at the upper axis limit for visualization.

Correlation analysis revealed distinct and separable compositional control over early dissolution, ion release, and antibacterial efficacy (Fig. 3A). Dissolution rate correlated positively with B₂O₃ (r ≈ 0.50) and negatively with CaO (r ≈ −0.57), indicating borate-driven network depolymerization moderated by calcium stabilization. Ag release rate showed a strong positive correlation with Ag₂O (r ≈ 0.93). Ca release rate correlated strongly with CaO (r ≈ 0.62). Antibacterial efficacy (% inhibition of *S. mutans*) correlated most strongly with Ag₂O (r ≈ 0.65). Ratio-based correlations further highlighted interaction effects, including B₂O₃:CaO with dissolution rate (r ≈ 0.62) and Ag₂O:NaF with % inhibition (r ≈ 0.54), supporting relative compositional balance and halide–silver interactions as key contributors to biological outcomes.

To integrate antibacterial performance with toxicological margin of safety, formulations were evaluated using a therapeutic window map (Fig. 3B). Several compositions achieving >50% inhibition fell below the safety threshold (MOS < 1), corresponding to effective but unsafe performance. A distinct subset of formulations satisfied both efficacy and safety criteria, defining a safe and effective therapeutic window. Within this region, calcium release rate spanned a broad range, allowing safety-compliant formulations to be distinguished based on calcium delivery within the defined therapeutic window.

## 4 Discussion

Dental caries continues to be one of the most widespread non-communicable diseases globally, best conceptualized as a biofilm-mediated, diet-induced dysbiosis resulting from frequent decreases in plaque pH following exposure to fermentable carbohydrates, rather than an acute infection model [1,5,62]. In this framework, toothpaste serves as a limited yet potent intervention medium: its exposure is brief and subject to dilution during brushing, yet it is applied daily, necessitating rapid efficacy at low delivered doses for antibacterial effects [63,64]. This study investigates borate-based bioactive glass formulations not as traditional antimicrobials or sustained-release agents, but as adaptable particles engineered for short-contact exposure, where early ion availability dictates biological effect and safety-by-design. By exploiting the composition-dependent dissolution and ion-release kinetics of borate glass networks, this strategy aims to contribute to diminishing reliance on conventional organic antimicrobials while integrating efficacy-focused design with antimicrobial stewardship considerations within a materials platform [24,25,65].

To reflect toothpaste-relevant exposure conditions, all compositions were evaluated under a clinically informed, fixed extraction regime applied consistently across the Design-of-Mixtures (DoM) space, rather than exaggerated extractables testing typical of ISO 10993 frameworks for medical devices. The extraction ratio was parameterized using clinically informed variables, including dentifrice mass per use, salivary flow during brushing, and exposure duration, to define a representative short-contact regime for consistent evaluation across compositions [13,66]. Maintaining this exposure condition within a DoM framework allows for the resolution of main and interaction effects across compositions while minimizing confounding variables [67,68]. This exposure-based approach is essential for preventive oral health technologies, where repeated, dilution-limited exposure is predominant, and stewardship-aligned design avoids excessive antimicrobial dosing [22].

Within this exposure context, a central contribution of this work is demonstrating that an ISO 10993-17 aligned toxicological risk assessment (TRA) can function as a primary, exposure-relevant, early-stage screening framework for rapidly dissolving, ion-releasing biomaterials intended for toothpaste applications [38]. By coupling brief exposure (∼2 min) with conservative assumptions regarding dentifrice mass, salivary dilution, ingestion (versus sustained local oral tissue contact), and daily use, the TRA directly evaluates the dominant safety-relevant mechanism in this system, systemic ion exposure, while composition and extract ratio remain active design variables [11,69]. Under these conditions, prolonged-extraction cytotoxicity assays developed for worst-case medical device evaluation may be less representative of real-world risk, and their omission reflects a deliberate staged-development strategy, with ISO 10993–5 cytotoxicity, biofilm-based assays, and in vivo models reserved for downstream validation following exposure-based screening [[70], [71], [72], [73]]. At the same time, this framework remains conservative and aligned to the intended brushing interval, daily use assumptions, and ingestion-dominated exposure pathway. Although longer-term accumulation and broader mixture effects remain relevant, these are more appropriately addressed during downstream product-level evaluation.

Evaluation of TRA outputs across ions indicates that boron and fluoride do not constrain systemic safety within the investigated design space. Modeled boron exposures remain well below the adult tolerable upper intake level, and fluoride exposures fall within adequate-intake-based tolerable exposure values even under conservative full-dissolution assumptions [3,51,53]. In contrast, silver consistently defines the upper boundary of systemic safety and emerges as the primary risk-driving ion. Following regulatory practice, a conservative tolerable exposure threshold based on the U.S. EPA reference dose (0.005 mg·kg^–1^•day^–1^; ∼0.35 mg/day for a 70-kg adult) was applied as the primary benchmark [56]. Under this conservative framework, formulations containing ≤0.75 mol% Ag_2_O maintain a margin of safety (MOS ≥ 1), whereas higher-silver compositions exceed the daily intake threshold under worst-case assumptions. To contextualize uncertainty and inter-individual variability, an alternative scenario incorporating published human observational data was also considered [[58], [59], [60]]. Under this permissive but evidence-based scenario, the modeled safety envelope expands to include compositions containing up to ∼1.5 mol% Ag_2_O under the same exposure assumptions. Together, these dual thresholds reflect toxicological practice in which benefit and risk lie on a continuum rather than a binary boundary.

Having established safety as the first gating criterion, early dissolution behavior provides the mechanistic context for interpreting efficacy. Under the clinically anchored, DoM-consistent extraction condition, all BAGF compositions exhibited substantial dissolution within the clinically relevant brushing window (mass loss ∼13–52%), corresponding to effective extract concentrations of ∼0.67–2.69 mg/mL. This variability reflects intrinsic, composition-governed early dissolution kinetics rather than experimental confounding and establishes early mass loss as the boundary condition governing ion availability during the brushing window [3,5]. Broader time-resolved dissolution and ion release behaviour across this glass series, including Ag, Ca, B, and F from 10 min to 24 h, were reported previously and are discussed there in greater detail [45].

Within this transient exposure regime, calcium release establishes a mechanistically meaningful link between composition and remineralization relevance. Calcium release was distributed within a narrow but biologically meaningful range, reflecting its dual role as a network modifier governing early dissolution and as the principal thermodynamic determinant of calcium phosphate stability [6,40,74]. In saliva and plaque fluid, where phosphate is typically non-limiting, calcium availability functions as a gating variable for remineralization permissiveness during short-duration exposure events [75,76]. Two-minute calcium release does not indicate mineral formation; rather, it confirms entry into a thermodynamically permissive regime for mineralization initiation. Consistent with this framing, mixture-response modeling reveals a strong CaO main effect and significant modifier interaction terms (including NaF) on early Ca release, demonstrating that glass composition governs both the magnitude and efficiency with which permissive conditions are established. Actual equations were used to predict response values at defined compositional levels, whereas coded equations were used to interpret the relative magnitude, direction, and interaction of component effects across the design space. Framing calcium release as a thermodynamic design constraint, rather than a biological endpoint, distinguishes this approach from remineralization studies reliant on prolonged immersion or pH-cycling assays and aligns with the clinical objective of repeatedly biasing daily demineralization–remineralization cycles toward recovery [3,77].

Within the safety-defined design space, antibacterial responses spanning bactericidal, bacteriostatic, and non-effective activity profiles were observed against *Streptococcus mutans*. Combined MIC/MBC outcomes, optical density (OD) kinetics, and mixture-response modeling demonstrate that antibacterial activity is governed by compositional interactions and extract ratio rather than extract concentration alone [78,79]. The divergence between OD suppression and bactericidal outcomes highlights a critical methodological consideration: OD kinetics reflect growth suppression but cannot confirm loss of viability without bactericidal endpoints, reinforcing the value of combined MIC/MBC evaluation for comparative screening across broad compositional spaces [[80], [81], [82]]. In the context of dental caries, where disease progression is driven by early biofilm establishment rather than acute infection, bacteriostatic responses may be clinically sufficient to suppress initial outgrowth while minimizing selective pressure for resistance [83].

Notably, these antibacterial activities were achieved using highly dilute extracts corresponding to only 25–50% of the 2 min dissolution products. Despite this dilution, early-time silver concentrations fell within therapeutic ranges widely reported to elicit MIC or MBC effects across oral and non-oral bacterial species (∼4–100 ppm) [[84], [85], [86]]. Importantly, several compositions failed to suppress growth despite releasing silver within these same ranges, demonstrating that antibacterial efficacy is not dictated by total silver availability alone. Instead, the response appears to be strongly composition-dependent, reflecting differences in dissolution behaviour, ion release kinetics, and possible local pH effects. This non-monotonic behavior contrasts with conventional silver-based antimicrobials designed for sustained or high-dose release and enables rational selection of compositions aligned with specific clinical objectives and exposure constraints [87,88].

Silver remains the dominant antibacterial contributor, consistent with its established mechanisms of membrane disruption, metabolic interference, and inhibition of DNA replication [41,42]. However, the ability to tune bacteriostatic versus bactericidal outcomes through composition rather than dose escalation embodies a resistance-conscious materials strategy particularly suited to preventive oral health applications characterized by repeated, low-level exposure [89,90].Within this framework, antibacterial behavior becomes a design variable rather than a fixed requirement. The observed non-linear mixture effects, particularly Ag₂O*NaF interaction terms, suggest an oligodynamic contribution in which halide species modulate silver speciation or bioavailability at low concentrations [91,92]. This interpretation is consistent with prior reports demonstrating anion-mediated enhancement of silver antimicrobial activity, in which halides increase efficacy at fixed or reduced total silver doses. The translational relevance of this interaction is amplified by fluoride’s established role in caries-preventive formulations, enabling halide–silver synergy to be leveraged within existing toothpaste architectures without introducing new antimicrobial classes [10,93].

Integrating safety, dissolution, antibacterial efficacy, and calcium thermodynamics into a single decision framework reveals a discrete therapeutic window in which meaningful antibacterial performance and acceptable toxicological margin coexist. As visualized in the therapeutic window map (Fig. 3B), antibacterial potency and toxicological acceptability are not intrinsically coupled, and formulations achieving high inhibition frequently fall outside the safety-acceptable region. From a materials design perspective, this outcome reflects an inherent exposure–response trade-off: while increasing Ag_2_O content or network solubility enhances delivered silver and antibacterial potency, it progressively erodes the margin of safety as tolerable intake thresholds are approached. Within the defined therapeutic window, antibacterial performance is therefore achieved through dose-efficient, compositionally mediated silver release rather than through maximal dosing. Notably, calcium release varies independently of antibacterial efficacy within this window, repositioning Ca^2+^ as a secondary but functionally important design variable linked to remineralization relevance rather than microbial killing.

This study is intentionally positioned as an exposure-anchored, early-stage screening effort rather than a complete biological validation package. Limitations include reliance on modeled adult exposure, absence of local irritation and long-term accumulation analyses, and use of planktonic antibacterial assays and calcium release as a thermodynamic proxy rather than direct mineral formation. A limitation of the OD-based antibacterial dataset is that replicate-level dispersion values were not provided by the external test facility, restricting formal interpretation of borderline visual differences in Fig. 2A; accordingly, antibacterial classifications were interpreted in conjunction with MIC/MBC outcomes and agar recovery rather than from OD bars alone. These simplifications are deliberate at this stage, enabling efficient resolution of composition–property relationships under realistic toothpaste-relevant exposure conditions. Guided by the therapeutic window identified here, future work should focus on down-selected formulations evaluated using biofilm-based antibacterial models, ISO 10993–5 cytotoxicity and irritation testing, and enamel-relevant mineralization assays under cyclic exposure, benchmarked against standard fluoride toothpaste formulations.

## 5 Conclusions

This work introduces a foundational safety-by-design framework for ion-releasing biomaterials in daily oral care, fundamentally shifting how preventive materials are developed under realistic exposure conditions. Integrating clinically relevant extraction with a Design-of-Mixtures approach, our framework embeds an ISO 10993-17–aligned toxicological risk assessment as a primary screen, enabling systemic safety and material performance to be evaluated concurrently. We show that early dissolution and multi-ion release are composition-governed processes that directly dictate functional outcomes on brushing-relevant timescales. Crucially, calcium release acts as a thermodynamic constraint defining remineralization permissiveness, while silver-mediated antibacterial efficacy is revealed as non-monotonic, driven by compositional interactions rather than dose escalation. These results definitively delineate a discrete therapeutic window: achieving robust antibacterial efficacy without compromising safety margins. By integrating exposure, safety, and ion interactions as co-equal design inputs, this work transforms preventive oral biomaterial development, moving beyond empirical screening toward rational, therapeutic design that explicitly couples efficacy with stringent safety constraints.

## CRediT authorship contribution statement

**Christine Andrea:** Writing – review & editing, Writing – original draft, Visualization, Validation, Resources, Project administration, Methodology, Investigation, Formal analysis, Data curation, Conceptualization. **Daniel Boyd:** Writing – review & editing, Supervision, Resources, Project administration, Methodology, Funding acquisition, Conceptualization.

## Declaration of competing interest

The authors declare the following financial interests/personal relationships which may be considered as potential competing interests: Daniel Boyd reports financial support was provided by Dalhousie Medical Research Foundation. Daniel Boyd reports financial support was provided by Early Stage Commercialization Fund (Invest Nova Scotia). Daniel Boyd reports financial support was provided by Natural Sciences and Engineering Research Council of Canada. If there are other authors, they declare that they have no known competing financial interests or personal relationships that could have appeared to influence the work reported in this paper.

## Data Availability

Data will be made available on request.
